# Model-free reconstruction of magnetic correlations in frustrated magnets

**DOI:** 10.1107/S2052252518006590

**Published:** 2018-06-01

**Authors:** Nikolaj Roth, Andrew F. May, Feng Ye, Bryan C. Chakoumakos, Bo Brummerstedt Iversen

**Affiliations:** a Center for Materials Crystallography (CMC), Department of Chemistry and Interdisciplinary Nanoscience Center (iNANO), Aarhus University, Langelandsgade 140, Aarhus 8000, Denmark; b Materials Science and Technology Division, Oak Ridge National Laboratory, Oak Ridge, TN 37831, USA; c Neutron Scattering Division, Oak Ridge National Laboratory, Oak Ridge, TN 37831, USA

**Keywords:** three-dimensional magnetic pair distribution function, magnetic correlations, frustrated magnets, magnetic diffuse scattering

## Abstract

Frustrated magnetic systems exhibit extraordinary physical properties, but quantification of their magnetic correlations poses a serious challenge to experiment and theory. Current insight into frustrated magnetic correlations relies on modelling techniques such as reverse Monte-Carlo methods, which require knowledge about the exact ordered atomic structure. Here, we present a method for direct reconstruction of magnetic correlations in frustrated magnets by three-dimensional difference pair distribution function analysis of neutron total scattering data. The methodology is applied to the disordered frustrated magnet bixbyite, (Mn_1−*x*_Fe_*x*_)_2_O_3_, which reveals nearest-neighbor antiferromagnetic correlations for the metal sites up to a range of approximately 15 Å. Importantly, this technique allows for magnetic correlations to be determined directly from the experimental data without any assumption about the atomic structure.

## Abstract 2

A new method for direct quantification of magnetic correlations in frustrated magnets is developed based on analysis of neutron total scattering data.

## 1. Introduction   

A perfect crystal is a three-dimensional object with complete long-range atomic order. Crystals containing magnetic atoms give rise to macroscopic magnetic properties, and indeed magnetic materials are essential to the function of modern society, being used extensively for information storage, electricity generation and in motors. Most of these materials have both long-range magnetic and atomic ordering, and their magnetic structures are quite well understood. However, advanced technologies will require more complex and even exotic magnetic phenomena, where atomically ordered materials do not possess long-range magnetic ordering. These disordered or frustrated magnetic materials include spin-glasses (Lee *et al.*, 1996 ▸, 2002 ▸; Paddison *et al.*, 2016 ▸), spin-liquids (Banerjee *et al.*, 2016 ▸, 2017 ▸), spin ice (Fennell *et al.*, 2009 ▸; Morris *et al.*, 2009 ▸), superconductors (Glasbrenner *et al.*, 2015 ▸; Tranquada *et al.*, 1996 ▸) and multiferroics (Jang *et al.*, 2017 ▸; Kalinin, 2017 ▸; Zhou *et al.*, 2007 ▸). Such materials only contain local short-range correlations in their magnetic structures; this makes it impossible to apply conventional experimental methods such as neutron diffraction, which is commonly used for studying long-range magnetism. Consequently, the progress on understanding and designing disordered spin systems has been hindered by the lack of adequate characterization of the local magnetic structure.

Magnetic disorder gives rise to fascinating phenomena, but in fact many crystals do not even contain three-dimensional atomic order. Atomic disorder in itself leads to a range of exciting properties, for example, atomic disorder strongly disrupts heat conduction in crystals. This has been used in numerous applications including the design of high performance thermoelectrics (Tan *et al.*, 2016 ▸).

Studies of atomic disorder represent a frontier of structural science and the recent introduction of the three-dimensional difference pair distribution function, 3D-ΔPDF, obtained from X-ray scattering on single crystals, has been a huge advance (Weber & Simonov, 2012 ▸). The 3D-ΔPDF method gives a three-dimensional view of only the disorder by eliminating contributions from the average ordered structure, which can be determined from conventional crystallographic methods. Unlike the frequently used one-dimensional PDF technique (Billinge & Egami, 2003 ▸), the 3D-ΔPDF method separates interactions at equal distances but different spatial directions; it also makes observation of weak disorder possible in systems with a superimposed average order. The 3D-ΔPDF thus provides information that cannot be obtained from other experimental techniques.

Similar to the analysis of structural disorder from diffuse X-ray scattering, diffuse magnetic neutron scattering can be used to gain insight into spin–spin correlations in magnetically disordered materials. Traditionally, this method has mainly relied on inspection of the wavevector and the temperature dependence of the scattering. Such an approach provides only a limited understanding of the disorder as reciprocal space analysis makes the interpretation of results challenging in terms of a real space physical model. Recently, more advanced methods have been developed, where modeling of the scattering pattern is done by reverse Monte-Carlo simulations for both powder and single-crystal data (Paddison *et al.*, 2013 ▸, 2016 ▸). In these methods, a model crystal is built and its structure is refined to obtain a good match between the calculated scattering pattern and the experimental data. Another recent approach has been the application of magnetic pair distribution function (mPDF) analysis for powder neutron scattering (Frandsen *et al.*, 2014 ▸; Frandsen & Billinge, 2015 ▸). Such analysis gives a one-dimensional representation of the pairwise magnetic interactions, both ordered and disordered. There are, however, at least two shortcomings of this one-dimensional technique. One being systems with an average magnetic order, but where there are local deviations. For such systems, the average order will dominate the mPDF and the disorder will be difficult to observe. Another case being systems where different pairwise interactions have similar distances leading to peak overlap in one-dimensional data. In such cases, it will be highly challenging to uniquely establish the magnetic structure. Here, we derive an expression for a three-dimensional magnetic difference pair distribution function (3D-mΔPDF). This function provides a model-independent three-dimensional reconstruction of magnetic disorder in real space. Since it does not rely on *a priori* information about the atomic structure, it allows studies of magnetism in both atomically and magnetically disordered materials, and indeed the combination of these may lead to the discovery of extraordinary new physical phenomena.

## 2. The three-dimensional magnetic difference pair distribution function   

The 3D-ΔPDF used for X-ray scattering is defined as the inverse Fourier transform of the scattered diffuse intensity, which is equal to the autocorrelation of the difference between the total electron density and the average periodic electron density, 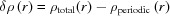 (Weber & Simonov, 2012 ▸): 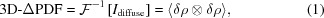
where 〈…〉 is the experiment time-average and 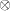 is the cross-correlation operator. Thus, the X-ray scattering 3D-ΔPDF only contains information about the atomic disorder, making it a powerful tool for establishing the local structure of disordered materials. The autocorrelation of the difference density will have positive peaks for vectors separating more electron density than in the average periodic structure and negative peaks for vectors separating less electron density than the average periodic structure.

Similar to the X-ray scattering 3D-ΔPDF, we define a 3D-mΔPDF as the inverse Fourier transform of the unpolarized magnetic diffuse neutron scattering cross-section
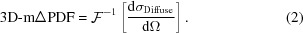
As the interaction potential for magnetic neutron scattering is a vector field and not a scalar field as for X-ray scattering, it is no longer simply the autocorrelation of a scalar density. We start our derivation by partitioning the magnetization density into an average periodic contribution and the deviations from it 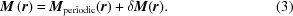
Note that in the case where there is no periodic magnetization density, 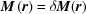. We wish to express the 3D-mΔPDF in terms of this difference magnetization density. In the supporting information, we show that, starting from standard equations (Lovesey, 1984 ▸), the 3D-mΔPDF can be written as: 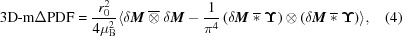
where we have defined the vector-field cross-correlation operator as a combination of element-wise cross correlation and a dot product: 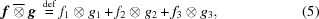
where 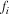 and 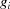 are the vector components of ***f*** and ***g***. Similarly, we have defined the vector-field convolution operator 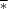 from the scalar field convolution, 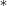. The smearing function that modifies the magnetization density in the second term is given by: 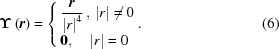



The first term in equation (4) is the vector autocorrelation of the difference magnetization density. Positive peaks in this function occur when the vector ***r*** separates more magnetization density pointing in the same direction than in the average periodic structure. Likewise, a negative peak occurs for vectors separating less magnetization density pointing in the same direction than in the average periodic structure. This can occur if either the magnetization direction is the same as the average, but less density is separated by the vector locally, or the density separated by ***r*** is oppositely aligned compared with the average structure. An important simplification occurs when there is no periodic magnetic structure (*e.g.* in frustrated magnets). In this case, a positive peak in the first term means that the magnetization density separated by ***r*** tends to be along the same direction, and a negative peak means the magnetization density separated by the vector is oppositely aligned.

The second term in equation (4) is less straightforward. The term originates from the fact that the scattering experiment only sees the magnetization density perpendicular to the scattering vector. A corresponding term was found by Frandsen *et al.* for the one-dimensional magnetic PDF (Frandsen *et al.*, 2014 ▸). In this term, the magnetization density is vector convoluted with the smearing function 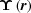 before the autocorrelation is taken. To get a better understanding of the effect of this second term, the 3D-mΔPDF for a number of simple systems is evaluated.

## 3. Simulations   

**Figure 1:**
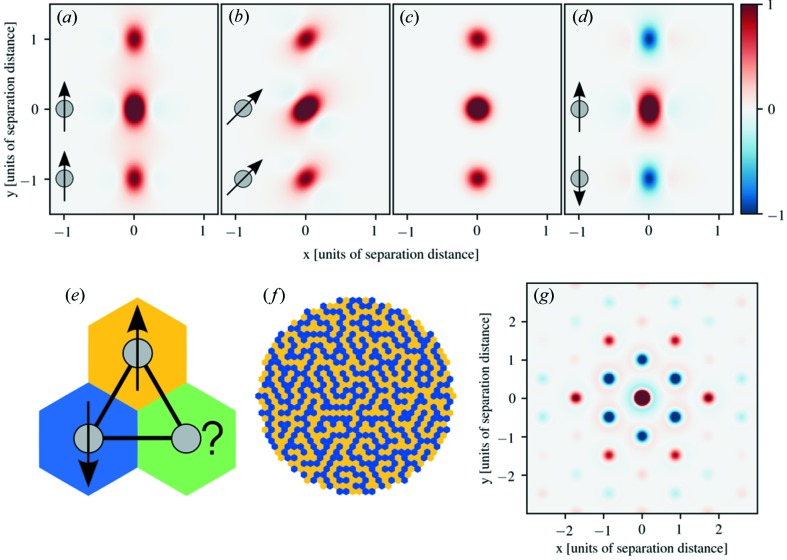
Simulations of the 3D-mΔPDF for simple systems. (*a*) Ferromagnetic alignment along the separating axis. A positive peak is always present at the origin as all magnetization density is aligned with itself. Positive peaks are also found at the separation vector, showing moments are aligned in same direction. (*b*) Ferromagnetic alignment tilted with respect to the separating axis. The 3D-mΔPDF is smeared in the direction of the moments. (*c*) Ferromagnetic alignment symmetry averaged for cubic symmetry. (*d*) Antiferromagnetically aligned moments. Negative peaks are found at the separation vector showing the opposite directions. (*e*) Antiferromagnetically coupled spins on a triangle. (*f*) A disordered ground state of the antiferromagnetic triangular Ising net (Wannier, 1950 ▸). Moments pointing into the plane are shown as blue and moments going out of the plane are yellow. It is calculated by starting with a random distribution of spin up/down, then repeatedly selecting a random spin and flipping it if it has more neighbors of the same type than opposite. (*g*) The 3D-mΔPDF for the antiferromagnetic triangular Ising net.

We first simulate the 3D-mΔPDF for a system with two localized magnetic moments, modeled by Gaussian densities, in cases where they are ferro- and antiferromagnetically coupled and aligned along different directions. Figs. 1 ▸(*a*), (*b*) and (*c*) show the 3D-mΔPDF for two moments aligned ferromagnetically. For these, as for all other 3D-mΔPDF maps, a positive peak is observed at the origin as all magnetization density is aligned with itself. Additional positive peaks are found at the separation vector between the two moments. This shows that the moments are aligned in the same direction. The difference between (*a*) and (*b*) is the tilt of the moments relative to the separation axis. A smearing is observed in the direction of the moments, coming from the second term in equation (4). In Fig. 1 ▸(*c*) the cubic symmetry average is shown for 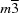 symmetry, and here, the direction dependent features of the moments are no longer seen because the positive and negative smearing features cancel. In Fig. 1 ▸(*d*), the 3D-mΔPDF for two antiferromagnetically aligned moments is shown; the negative peaks are found at the separation vector, showing the opposite alignment of moments.

The 3D-mΔPDF method is expected to be especially useful for systems with frustrated magnetism, which occurs when the magnetic moments in a structure are prohibited from having all preferences for correlations fulfilled. A simple example of this is three moments in a triangle with antiferromagnetic coupling, as shown in Fig. 1 ▸(*e*). For such a system, it is only possible to satisfy two of the three interaction preferences. Similarly, the antiferromagnetic triangular Ising net will adapt a disordered ground state, as it is not possible for all moments to be neighboring moments of opposite direction, shown by Wannier (1950 ▸). An example of one such ground state is illustrated in Fig. 1 ▸(*f*). In Fig. 1 ▸(*g*) we show the corresponding 3D-mΔPDF. The vectors for the nearest neighbor interactions show negative peaks, indicating the preference for antiferromagnetic alignment. Similarly, the next-nearest neighbor vectors show positive peaks, suggesting that these spins tend to align in the same direction. From the features of the 3D-mΔPDF, information about the relative orientation of magnetic moments can thus be observed directly. The interpretation of the peaks is the same as for the first term in equation (4), keeping in mind that features are smeared out as a result of the second term.

## 4. Experimental determination of the 3D-mΔPDF   

**Figure 2:**
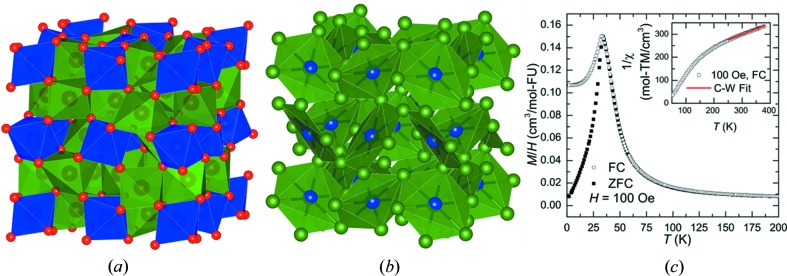
Structure and magnetization of bixbyite. (*a*) Polyhedral model of bixbyite, where the *M*1 octahedra are shaded blue and the *M*2 polyhedra are green. The red spheres are oxygen atoms each tetrahedrally coordinated by *M*. The octahedra share corners and edges to make a three-dimensional framework. (*b*) *M*-only atoms of bixbyite showing the near-neighbors of the *M*1 sites (blue) surrounded by the *M*2 sites (green). Nearly perfect hexagons of *M*1(*M*2)_6_ result and share corners to make a three-dimensional cubic network. (*c*) Field-cooled (FC) and zero-field- cooled (ZFC) magnetization data for bixbyite. The inset shows the 1/χ behavior, the red line indicating the Curie–Weiss fit.

To demonstrate the strength of our new method, we study the magnetic disorder in the naturally occurring mineral bixbyite, (Mn^3+^,Fe^3+^)_2_O_3_, which has the β-Mn_2_O_3_ crystal structure (cubic, 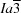, *a* = 9.41 Å) (Pauling & Shappell, 1930 ▸). This crystal structure has triangular and hexagonal arrangements of near-neighbor metal sites *M*1 and *M*2, as seen in Fig. 2 ▸(*b*), suggesting the possibility of magnetic frustration. The naturally occurring crystal used for this study is of the composition Fe_1.1_Mn_0.9_O_3_, determined by both neutron diffraction and ICP measurements (see supporting information). The Fe and Mn atoms are disordered over the two metal sites in the structure. From magnetization measurements, it is found that a transition occurs at T* = 32.5 K, as seen in the cusp of Fig. 2 ▸(*c*), where temperature-dependent magnetization data are shown for field-cooled (FC) and zero-field-cooled (ZFC) measurements. The inset in Fig. 2(*c*) shows 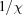 plotted as a function of temperature and the red line shows the region where a Curie–Weiss law is obeyed. The data clearly reveal a negative Weiss temperature, indicating that antiferromagnetic interactions are dominant in the paramagnetic phase at high temperatures. To understand the low-temperature magnetic phase, single-crystal neutron scattering data were collected. The nuclear structure is identical at all temperatures in the range 7–300 K, and there is no sign of long-range magnetic ordering. To verify the lack of long-range magnetic ordering, we have measured time, temperature and field-dependent DC magnetization, ac magnetic susceptibility and specific heat capacity, as shown in the supporting information. These measurements support the treatment of bixbyite Fe_1.1_Mn_0.9_O_3_ as a phase without long-range magnetic order, and T* is found to be associated with a spin-glass transition.

As there is no long-range magnetic order on the metal sites in the low-temperature phase of bixbyite, the resulting 3D-mΔPDF will be straightforward to interpret. Because 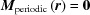 then 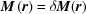, so the resulting 3D-mΔPDF will contain information about the whole magnetization density. Furthermore, as the system has cubic symmetry, the spurious effect arising from the second term in equation (4) will cancel, as shown by simulations in Fig. 1 ▸(*c*). Positive and negative features in the 3D-mΔPDF can then be directly interpreted in terms of magnetic moments preferring parallel and antiparallel alignment, respectively.

**Figure 3:**
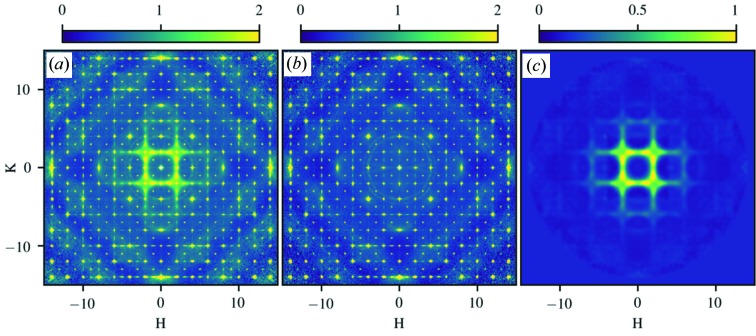
Reciprocal space neutron scattering for bixbyite. All figures are of the *HK*0 plane. (*a*) Total elastic scattering at 7 K. (*b*) Total elastic scattering at 300 K. (*c*) Isolated magnetic diffuse scattering.

To produce an experimental 3D-mΔPDF, the magnetic diffuse neutron scattering has to be known. We have measured the elastic unpolarized neutron scattering at 7 and 300 K, where one temperature is above the transition (*i.e.* the paramagnetic regime) and the other is below the transition (*i.e.* in the disordered spin-glass regime). These data were collected at the CORELLI spectrometer at the Spallation Neutron Source at Oak Ridge National Laboratory (Rosenkranz & Osborn, 2008 ▸). CORELLI’s design enables elastical discrimination of the total scattering, *i.e.* the phonon and thermal diffuse scattering are removed. From the two data sets the full elastic reciprocal space-scattering intensities are reconstructed using the Laue symmetry of the crystal. The reconstructed *HK*0 plane at the two temperatures can be seen in Figs. 3 ▸(*a*) and (*b*). Since the nuclear structure is identical at 7 and 300 K, the data from the paramagnetic regime can be subtracted from the low-temperature data to remove all scattering contributions other than magnetic scattering. This includes nuclear scattering, both Bragg and diffuse, as well as background scattering. After the subtraction, residual errors are present at the position of the very sharp Bragg peaks. To remove these, a punch-and-fill method is employed, where a small volume around each reflection is removed and filled with a smooth function to resemble the diffuse scattering in that region (see the supporting information). In cases where there would be a long-range magnetic ordering, the same punch-and-fill method would be used to remove the magnetic Bragg scattering. The high-angle data are also removed, as they mainly consist of noise. The result of this process is the isolated magnetic diffuse scattering in three-dimensional reciprocal space. Diffuse magnetic scattering for the *HK*0 plane from this processing of the bixbyite data can be seen in Fig. 3 ▸(*c*). A more detailed description of the data reduction process can be found in the supporting information.

**Figure 4:**
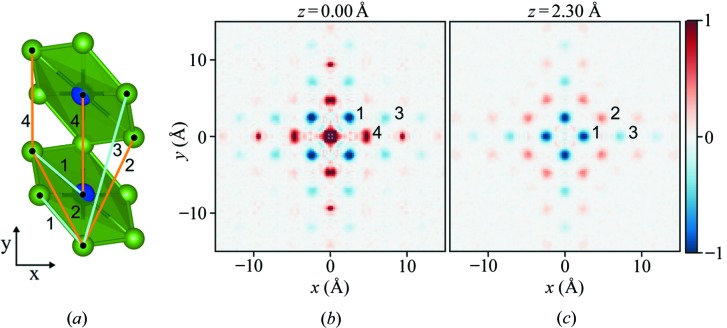
The 3D-mΔPDF for bixbyite. (*a*) Selected portion of the structure showing numbered vectors between atoms. (*b*) 3D-mΔPDF for the z = 0 plane. (*c*) 3D-mΔPDF for the z = 2.30 Å plane. The slight split of peak number 4 is an artefact, as the vector should have a component along only one axis.

The 3D-mΔPDF is then simply obtained by Fourier transformation. Two planes of the 3D-mΔPDF for bixbyite are shown in Figs. 4 ▸(*b*) and (*c*). As there is no long-range periodic magnetic order, the peaks in the 3D-mΔPDF can be directly interpreted as the alignment preference between sites separated by the corresponding vector. A few of the features in the maps have been marked with numbers for which the corresponding vectors in the crystal structure are shown in Fig. 4 ▸(*a*). The nearest-neighbor vector (marked 1), which is for both site pairs *M*1–*M*2 and *M*2–*M*2, has a negative peak in the 3D-mΔPDF, which identifies that nearest-neighbor metal sites tend to have antiferromagnetic alignment. The 3D-mΔPDF for the vectors for the next-nearest neighbor pairs (marked 2) is positive, showing a preference for alignment in the same direction. As both of the metal sites in the structure contain disordered mixtures of Fe and Mn, the local magnetic structure could be expected to be very complicated, depending on local distributions of Fe and Mn on the two sites. However, using the 3D-mΔPDF technique we observe that on average, the metal sites have an antiferromagnetic nearest-neighbor correlation. These correlations can then be followed to longer distances, showing alternating positive and negative peaks for higher order neighbors. Overall, the 3D-mΔPDF for bixbyite clearly shows the disordered low-temperature state to be dominated by an antiferromagnetic nearest-neighbor interaction. Peaks in the 3D-mΔPDF fall off rapidly and disappear after ∼15 Å, directly revealing the maximum distance of the magnetic correlations, which is not greater than two unit cells. The 3D-mΔPDF results are expectedly self-consistent with the field- and temperature-dependent magnetization measurements, but, moreover, directly show the 3D atomic pairwise correlations that exist in the frozen spin state, without making any assumptions about the system.

## 5. Discussion   

The 3D-mΔPDF has two major advantages compared with the 1D-mPDF introduced by Frandsen *et al.* (Frandsen *et al.*, 2014 ▸). One is for systems with average long-range magnetic order and local deviations. In this case, the 3D-mΔPDF will show the deviations from average structure directly, and the average magnetic structure can then be found by separate analysis of Bragg reflections. In such systems, the 1D-mPDF superimposes the ordered and disordered parts of the structure, making it difficult to interpret the disorder which is often a small deviation from the average structure. The second advantage arises from the fact that the magnetic scattering falls off rapidly in reciprocal space as the electrons responsible for the magnetic moment are diffuse. This affects the broadness of peaks in the PDF functions during Fourier transform. For the 1D-mPDF, the peak broadness can easily lead to overlap of neighboring peaks, once again making the interpretation less straightforward. Peak overlap is also obtained when multiple interactions have the same distance but different spatial directions. In such cases, the 3D-mΔPDF retains the directional information, making it possible to separate peaks close or equal in distance, but with different directions. However the issue of the peak broadness in the 1D-mPDF can be reduced by normalizing the data with the average magnetic scattering factor for the magnetic scatterers in the sample. In addition to this, refinement methods for the 1D-mPDF have been established, making it possible to regain the three-dimensional information through modelling (Frandsen & Billinge, 2015 ▸). As the 1D-mPDF comes from measurements of powders, it can be used to study a broad range of samples, whereas the 3D-mΔPDF requires large high-quality single crystals.

In the bixbyite system we were able to separate the magnetic diffuse scattering from nuclear diffuse scattering and scattering from the sample environment by subtracting a high-temperature dataset of the same structure. In cases where there is a structural transition between the paramagnetic and frustrated magnetic states, this method cannot be used. In cases where there is a structural change, but the low-temperature structure is ordered (no nuclear diffuse scattering), the magnetic diffuse scattering can be isolated by subtracting the scattering from an empty sample environment and using the punch-and-fill method on the Bragg peaks. In cases where there is a structural transition to a disordered structure, the magnetic diffuse scattering can be potentially isolated by polarized neutron scattering. Here we used the elastic discrimination of the CORELLI instrument to remove phonon scattering from the data, as this contribution is different at 300 and 7 K. The effect of only using the elastic scattering on the 3D-mΔPDF is that only the static magnetic correlations are seen; this means that magnetic excitations such as magnons are not seen. For bixbyite this is not a problem, as the magnetic diffuse scattering is elastic (see supporting information), but in cases where the dynamical magnetic correlations are wanted, the energy-integrated signal should be used for producing the 3D-mΔPDF. This is further discussed in the supporting information.

In conclusion, we have derived an expression for the 3D-mΔPDF, which directly reveals magnetic correlations for systems with disordered magnetism. Since it is a direct-space function, an intuitive interpretation is easily obtained which provides a better understanding of magnetic disorder, even for complex systems. Unlike previous studies of disordered magnetic systems, this new method is completely model independent. As the 3D-mΔPDF is simply the Fourier transform of the magnetic diffuse scattering, it provides a direct space view of all information about the magnetic disorder contained in the scattering data. In contrast to reverse Monte-Carlo models previously used for interpretation of magnetic diffuse neutron scattering, the 3D-mΔPDF is not challenged by false minima, although this can be mitigated by repeated simulations with randomized starting conditions. More importantly, the 3D-mΔPDF approach does not require a specific structural model, and this makes it possible to also study magnetism in atomically disordered systems such as bixbyite. The end members of bixbyite, Mn_2_O_3_ and β-Fe_2_O_3_, are known to go through phase transitions to ordered magnetic phases (Cockayne *et al.*, 2013 ▸; Malina *et al.*, 2015 ▸). This suggests that the presence of atomic disorder allows tuning of this complex magnetic system to create the magnetic frustration described above.

## 6. Related literature   

The following references are cited in the supporting information: Balanda (2013 ▸); Binder & Young (1986 ▸); Kobas *et al.* (2005 ▸); Mydosh (1993 ▸); Robitaille *et al.* (2013 ▸); Sheldrick (2001 ▸; 2008 ▸); Ye *et al.* (2018 ▸).

## Supplementary Material

Supporting information. DOI: 10.1107/S2052252518006590/yu5013sup1.pdf
