# Supplementary material for: Model-free reconstruction of magnetic correlations in frustrated magnets
Source: IUCrJ. 2018 Jun 1;5(Pt 4):410–6. doi: 10.1107/S2052252518006590 (PMC6038962; doi:10.1107/S2052252518006590)
Supplement: Supplementary file 1 [file m-05-00410-sup1.pdf]

IUCrJ

Volume 5 (2018)

Supporting information for article:

Model-free reconstruction of magnetic correlations in frustrated magnets

Nikolaj Roth, Andrew F. May, Feng Ye, Bryan C. Chakoumakos and Bo Brummerstedt Iversen

S1.

S2. Materials and Methods

S2.1. Physical property measurements

Using a single crystal, DC magnetization measurements were performed in a Quantum Design Magnetic Property Measurement System (MPMS), and ac magnetic susceptibility and specific heat capacity measurements were performed in a Quantum Design Physical Property Measurement System (PPMS). The ac magnetization measurements were performed with zero applied dc field, using amplitude of A_{AC} = 14Oe at various frequencies *f*. Time-dependent measurements were performed in the MPMS by ramping the magnetic field from 0 to 1000 Oe at various temperatures after the sample was taken to 50K or higher and cooled in zero applied field. The quantity M_0 is defined as the first measurement after the applied field of 1000 Oe was stabilized, for a given temperature; note that it took roughly 150s for the field to stabilize and the origin t=0 is defined by the first measurement M_0 not the time when H=0.

The physical properties results are shown in figure S1. The magnetization data of Bixbyite, shown in figure S1a, are characterized by a high-temperature paramagnetic region, and a cusp at T*=32.5K that at first glance would appear to be associated with antiferromagnetic ordering. Below T*, we observe a large divergence between the zero-field cooled (warming) data and the field-cooled data, which is expected for spin-glasses (Binder & Young, 1986). To further examine this behavior, we performed specific heat capacity measurements, shown in figure S1c, and did not observe any anomaly in the specific heat at T*. AC magnetic susceptibility measurements and time-dependent dc measurements were performed to establish the presence of glassy dynamics of the spins in Bixbyite. The maximum in the in-phase component of the ac susceptibility, χ' shown in figure S1b, clearly shifts downward in magnitude and to higher temperature as the frequency *f* increases, which is the behavior expected for a spin glass (Binder & Young, 1986, Mydosh, 1993, Balanda, 2013). Consistent with the behavior of χ' for a spin-glass, we observe a time-dependence of the dc magnetization data at T = 2 and 30K, but not at 50K, which is shown in figure S1d. Therefore, T* appears to be associated with a spin freezing transition and these results support the treatment of Bixbyite Fe1.1Mn0.9O3 as a phase that does not possess long-range order.

In the insert in figure S1a, $1/\chi$ is plotted as a function of temperature. Above 200K, the DC susceptibility $\chi = \frac{M}{H}$ is found to follow the Curie-Weiss law $\chi = \frac{C}{T-\Theta}$, where C is the Curie constant related to the effective moment and Θ the Weiss temperature. For an applied field of H=100 Oe, we fit the data between 200 and 380K and obtained an effective moment of $\frac{4.1(1)\mu_B}{TM}$, where TM = transition

metal. The data also clearly reveal a negative Weiss temperature, with the fit yielding $\Theta = -336(2)K$. The negative Weiss temperature indicates antiferromagnetic interactions are dominant in the paramagnetic phase, consistent with the spin-spin correlation information obtained from the 3D-mΔPDF at lower temperatures. The frustration ratio, $|\Theta|/T^* = 336/32.5=10.3$ demonstrates a significant amount of frustration in this natural bixbyite sample. The DC M/H data show a strong deviation from Curie-Weiss behavior below approximately 150K. From the data, it appears that M/H is being enhanced ($\frac{1}{\chi}$ is reduced below the paramagnetic expectation). This may suggest some ferromagnetic component associated with the short-range order, such as canting of the local AFM order.

S2.2. Scattering and production of 3D-mΔPDF

Single crystal elastic neutron scattering was measured at the CORELLI beamline at the Spallation Neutron Source at Oak Ridge National Laboratory (Rosenkranz & Osborn, 2008). A piece was cut from a large cubic crystal, and subsequently sanded down to a sphere to limit crystal shape effects in the scattering. The crystal was glued to the end of an aluminum pin which was wrapped in neutron absorbing Cd-foil and mounted in a low background closed-cycle refrigerator. Measurements were carried out at 7K, 25K, 50K, 80K, 160K, 240K and 300K, with the 7K and 300K being longer for increased counting statistics. Background scattering for subtraction was measured on an aluminum pin with Cd foil, but no crystal.

For every rotation angle of the crystal, the UB matrix was determined, as it was found that small errors were present in the goniometer rotation angle. Elastic-only scattering intensities in reciprocal space were reconstructed from the data using the UB matrices and normalized to vanadium flux. Symmetrization with the Laue symmetry (*m-3*) was employed in the reconstruction to fill out reciprocal space and to increase counting statistics. If the intensities were to be used for the production of a 3D-mΔPDF, no background subtraction was employed, as it would be subtracted later. For the production of an intensity map, background scattering was subtracted. Scattered intensities were reconstructed on a 501*501*501 point grid with each axis spanning between +-13.67$Å^{-1}$.

The production of a 3D-mΔPDF is done in the following way: Intensities measured at 300K, well into the paramagnetic regime, are subtracted from intensities measured at a lower temperature. In this way, all scattering contributions other than magnetic scattering are approximately removed. This includes nuclear scattering, both Bragg and diffuse, as well as background scattering. While the broad nuclear diffuse scattering and background scattering are well subtracted, the very sharp Bragg scattering has some residual errors. To remove these, a punch and fill method is employed. A similar method was described in (Kobas *et al.*, 2005). The method used here is slightly different from the one described by Kobas..

Instead of using rectangular boxes as the punch, the closest approximation to an ellipsoid on a rectangular grid is used. In this case where a cubic grid is used for the scattered intensities, the closest approximation to a sphere is used. As the Bragg peaks are close to being spherical in reciprocal space, this type of punch allows for complete removal of peaks while removing as little as possible from the rest of the scattered intensity. Strong reflections were punched with a sphere of 7 pixels in diameter, while weak reflections were punched with spheres of 3 pixels diameter. The intensities are punched by setting their values to NaN. The punched intensities then need to be filled in by a smooth function, resembling the diffuse scattering at the Bragg peaks. We accomplish this using the astropy convolution function for Python (Robitaille *et al.*, 2013). This function allows for convolution of 3D arrays where NaN values are replaced with interpolated values using the kernel as an interpolation function. In this case we use a Gaussian with a 2 pixel standard deviation as a kernel. By the convolution function used to fill in the punched intensities, the whole scattered intensity is also smoothed slightly, as seen in Figure 3. Regions of reciprocal space where no or very small amounts of data had been measured were removed. This includes the far reciprocal space where $\sqrt{h^2+k^2+l^2} > 14$, the corners where h,k,l are all larger than 4.5 and a sphere of diameter 11 pixels in the center of reciprocal space, as the direct beam is not measured. Through this process, the magnetic diffuse scattering has been isolated. The process is illustrated in Fig. S2. Figure S2A and B show the elastic neutron scattering at 7K and 300K, respectively. Figure S2C shows the difference when subtracting the 300K data from the 7K data. Figure S2D shows the difference scattering with holes at the Bragg positions as well as removed high angle noise. Figure S2E shows the finished isolated magnetic diffuse scattering after the filling process.

The punch and fill method used here will have two effects on the 3D-mΔPDF, one of which is easily removed. As the diffuse scattering intensity at the Bragg positions is filled by a smooth function, some error is introduced. However as these errors will be in the high-frequency components of the intensity, it will only affect the 3D-mΔPDF for long distances. But as we are only concerned with short-range interactions in the 3D-mΔPDF, the error introduced by this is not critical. The second effect comes from the convolution of the whole scattering pattern with a Gaussian. When the array is then Fourier transformed, the resulting array will correspond to the product of the 3D-mΔPDF with the Fourier transform of the Gaussian kernel, as required by the convolution theorem. The effect of the Gaussian kernel is then simply removed by dividing the array by the Fourier transform of the Gaussian, which is known, and thereby obtaining the 3D-mΔPDF.

When subtracting the high temperature dataset from the low temperature data to remove the nuclear and background contribution from the scattering, a paramagnetic scattering signal is also subtracted. In the paramagnetic regime, the spins are not correlated and the resulting scattering is simply proportional to the single-atom scattering factor. This is very slowly varying and isotropic in reciprocal space (Lovesey, 1984). The result of this subtraction is that slightly negative values are found in the inner

region of reciprocal space, as can be seen in Figure S2C. When Fourier transforming to the 3D-mΔPDF this only affects the r=0 signal and not the correlations which we are interested in.

Bragg peaks were integrated using an instrument specific script, corrected for absorption and merged using XPREP (Sheldrick, 2001) to *m*-3 symmetry. The structure was refined using SHELXL (Sheldrick, 2008) in the space group *Ia*-3. Both metal sites were refined as a combination of Mn and Fe with the constraint that each site is fully occupied. The twin law [1, 0, 0; 0, 0, 1; 0, 1, 0] was used during refinement. In Table T1 refinement results are shown.

As the fit is good, and approximately equally good at all temperatures is seems that the nuclear structure is sufficient to model the Bragg reflections. This suggests that there is no long-range magnetic ordering at low temperatures, which agrees well with the physical property measurements. To further test this, we have followed the reflection intensities with temperature. No new reflections are observed when cooling the sample, so any long-range magnetic structure would have to be contained in the existing Bragg reflections. In Figure S3 we have plotted a number of normalized low order reflection intensities with temperature, as any magnetic structure is expected to be seen in the low order region.

No significant changes in intensities are found when cooling the sample. The scattering data thus indicate that there does not seem to be any long-range magnetic ordering in the sample based on three points. These points are: 1) No appearance of new diffraction peaks when cooling the sample. 2) No significant changes in low order reflection intensities. 3) The nuclear structure describes the data well without the need for any magnetic model.

To check the composition found from the neutron diffraction data we have measured Inductively Coupled Plasma Atomic Emission Spectroscopy (ICP-EAS). From this we get then Fe/Mn ratio to be 1.15(1):0.85(1), which is close to the values found from refinement of diffraction data.

S3. Supplemantary Text

S3.1. Derivation

As can be found in most texts on magnetic diffuse neutron scattering, e.g. (*20*), the unpolarized cross-section for a magnetic system in the static approximation can be written as:

$$\frac{d\sigma}{d\Omega} = r_0^2 \langle \boldsymbol{Q}_\perp(-\boldsymbol{k}) \cdot \boldsymbol{Q}_\perp(\boldsymbol{k}) \rangle \qquad \text{[S1]}$$

Where $r_0 = \frac{\gamma e^2}{m_e c^2}$, $\boldsymbol{k}$ is the scattering vector, $\langle\,\rangle$ is the time average of the experiment and $\boldsymbol{Q}_\perp$ is the Fourier transform, denoted $\mathcal{F}$, of the magnetization density perpendicular to $\boldsymbol{k}$**:**

$$\boldsymbol{Q}_\perp(\boldsymbol{k}) = -\frac{1}{2\mu_B} \int d\boldsymbol{r} \exp(\mathrm{i}\mathbf{k}\cdot\mathbf{r})\, \tilde{\boldsymbol{k}} \times \left(\boldsymbol{M}(\boldsymbol{r}) \times \tilde{\boldsymbol{k}}\right) = -\frac{1}{2\mu_B} \mathcal{F}\left[\tilde{\boldsymbol{k}} \times \left(\boldsymbol{M}(\boldsymbol{r}) \times \tilde{\boldsymbol{k}}\right)\right] \qquad \text{[S2]}$$

Here $\boldsymbol{r}$ is the real-space vector and $\widetilde{\boldsymbol{k}} = \boldsymbol{k}/|\boldsymbol{k}|$ is the unit vector in reciprocal space. $\boldsymbol{M}(\boldsymbol{r})$ is the total magnetization density, the sum of both spin and orbital magnetization densities. Another way to partition the magnetization density is into an average periodic structure without disorder and the deviations from it

$$\boldsymbol{M}(\boldsymbol{r}) = \boldsymbol{M}_{periodic}(\boldsymbol{r}) + \delta\boldsymbol{M}(\boldsymbol{r}) \qquad [S3]$$

The term in the cross-section arising from the periodic magnetization density will give rise to sharp Bragg peaks in reciprocal space. The scattering from the deviations from the average periodic structure we call the magnetic diffuse scattering, and is what were are interested in here. There will also be a third cross term in the scattering cross section between the average structure and deviations from it. This term will only exist on the reciprocal lattice points, but will be much smaller than the Bragg scattering from the average periodic structure, and is therefore usually neglected.

We wish to arrive at an expression for the inverse Fourier transform of the magnetic diffuse scattering cross-section. We first insert equation [S2] for the deviations from periodic structure into equation [S1] and rearrange:

$$\frac{d\sigma_{Diffuse}}{d\Omega} = \frac{r_0^2}{4\mu_B^2}\langle \mathcal{F}\left[\widetilde{\boldsymbol{k}} \times \left(\delta\boldsymbol{M}(\boldsymbol{r}) \times \widetilde{\boldsymbol{k}}\right)\right]^* \cdot \mathcal{F}\left[\widetilde{\boldsymbol{k}} \times \left(\delta\boldsymbol{M}(\boldsymbol{r}) \times \widetilde{\boldsymbol{k}}\right)\right]\rangle \qquad [S4]$$

Where * denotes the complex conjugate. As the Fourier transform is an integral with respect to $\boldsymbol{r}$, then $\widetilde{\boldsymbol{k}}$ is a constant vector in regards to the transform. In this case, it can then easily be shown that

$$\mathcal{F}\left[\widetilde{\boldsymbol{k}} \times \left(\delta\boldsymbol{M}(\boldsymbol{r}) \times \widetilde{\boldsymbol{k}}\right)\right] = \widetilde{\boldsymbol{k}} \times \left(\mathcal{F}[\delta\boldsymbol{M}(\boldsymbol{r})] \times \widetilde{\boldsymbol{k}}\right) \qquad [S5]$$

By using the identity for the triple cross product and that $\widetilde{\boldsymbol{k}} \cdot \widetilde{\boldsymbol{k}} = 1$ we get:

$$\widetilde{\boldsymbol{k}} \times \left(\mathcal{F}[\delta\boldsymbol{M}(\boldsymbol{r})] \times \widetilde{\boldsymbol{k}}\right) = \mathcal{F}[\delta\boldsymbol{M}(\boldsymbol{r})] - \widetilde{\boldsymbol{k}}\left(\mathcal{F}[\delta\boldsymbol{M}(\boldsymbol{r})] \cdot \widetilde{\boldsymbol{k}}\right) \qquad [S6]$$

Inserting this into equation [S4] and reducing gives:

$$\frac{d\sigma_{Diffuse}}{d\Omega} = \frac{r_0^2}{4\mu_B^2}\langle \mathcal{F}[\delta\boldsymbol{M}(\boldsymbol{r})]^* \cdot \mathcal{F}[\delta\boldsymbol{M}(\boldsymbol{r})] - \left(\mathcal{F}[\delta\boldsymbol{M}(\boldsymbol{r})] \cdot \widetilde{\boldsymbol{k}}\right)^* \cdot \left(\mathcal{F}[\delta\boldsymbol{M}(\boldsymbol{r})] \cdot \widetilde{\boldsymbol{k}}\right)\rangle \qquad [S7]$$

We now define the three-dimensional magnetic difference pair distribution function as the inverse Fourier transform of the magnetic diffuse scattering cross-section:

$$3D - m\Delta PDF = \mathcal{F}^{-1}\left[\frac{d\sigma_{Diffuse}}{d\Omega}\right] \qquad [S8]$$

To simplify expressions, we need to consider how to treat inverse Fourier transforms of dot products. We are looking for an analogue to the cross-correlation theorem. The cross correlation, $\otimes$, of two scalar fields, $f(\boldsymbol{k})$ and $g(\boldsymbol{k})$ is related to the convolution operator, $*$, by:

$$f(\boldsymbol{k}) \otimes g(\boldsymbol{k}) = f^*(-\boldsymbol{k}) * g(\boldsymbol{k})$$

For scalar fields, the convolution and cross-correlation theorems say:

$\mathcal{F}[f \otimes g] = \mathcal{F}[f]^* \cdot \mathcal{F}[g]$ and $\mathcal{F}[f * g] = \mathcal{F}[f] \cdot \mathcal{F}[g]$

If we define two vector fields

$$\boldsymbol{f}(\boldsymbol{k}) = \begin{pmatrix} f_1(\boldsymbol{k}) \\ f_2(\boldsymbol{k}) \\ f_3(\boldsymbol{k}) \end{pmatrix} \text{ and } \boldsymbol{g}(\boldsymbol{k}) = \begin{pmatrix} g_1(\boldsymbol{k}) \\ g_2(\boldsymbol{k}) \\ g_3(\boldsymbol{k}) \end{pmatrix}$$

Then the inverse Fourier transform of the dot product of $\boldsymbol{f}^*$ and $\boldsymbol{g}$ will give

$$\mathcal{F}^{-1}[\boldsymbol{f}^* \cdot \boldsymbol{g}] = \mathcal{F}^{-1}[f_1^* g_1] + \mathcal{F}^{-1}[f_2^* g_2] + \mathcal{F}^{-1}[f_3^* g_3]$$

$$= \mathcal{F}^{-1}[f_1] \otimes \mathcal{F}^{-1}[g_1] + \mathcal{F}^{-1}[f_2] \otimes \mathcal{F}^{-1}[g_2] + \mathcal{F}^{-1}[f_3] \otimes \mathcal{F}^{-1}[g_3]$$

If we then define the vector cross correlation operator, $\overline{\otimes}$, such that

$$\boldsymbol{f} \,\overline{\otimes}\, \boldsymbol{g} \stackrel{\text{def}}{=} f_1 \otimes g_1 + f_2 \otimes g_2 + f_3 \otimes g_3$$

Then we have the cross-correlation theorem for vector fields as:

$$\mathcal{F}^{-1}[\boldsymbol{f}^* \cdot \boldsymbol{g}] = \mathcal{F}^{-1}[\boldsymbol{f}] \,\overline{\otimes}\, \mathcal{F}^{-1}[\boldsymbol{g}] \quad \Leftrightarrow \quad \mathcal{F}[\boldsymbol{f}]^* \cdot \mathcal{F}[\boldsymbol{g}] = \mathcal{F}\left[\boldsymbol{f} \,\overline{\otimes}\, \boldsymbol{g}\right] \qquad \text{[S9]}$$

Similarly, this can be done to define the convolution operator $\overline{*}$, giving a vector convolution theorem.

We are now ready to return to the problem at hand, which is the inverse Fourier transform of the scattering cross- section. There are two terms in the expression. The first is of the form:

$$\mathcal{F}^{-1}\left[\mathcal{F}[\delta\boldsymbol{M}(\boldsymbol{r})]^* \cdot \mathcal{F}[\delta\boldsymbol{M}(\boldsymbol{r})]\right] = \delta\boldsymbol{M}(\boldsymbol{r}) \,\overline{\otimes}\, \delta\boldsymbol{M}(\boldsymbol{r}) \qquad \text{[S10]}$$

This term is the vector autocorrelation of the disorder magnetization density. The second term is of the form:

$$\mathcal{F}^{-1}\left[\left(\mathcal{F}[\delta\boldsymbol{M}] \cdot \tilde{\boldsymbol{k}}\right)^* \cdot \left(\mathcal{F}[\delta\boldsymbol{M}] \cdot \tilde{\boldsymbol{k}}\right)\right] = \mathcal{F}^{-1}\left[\mathcal{F}[\delta\boldsymbol{M}] \cdot \tilde{\boldsymbol{k}}\right] \otimes \mathcal{F}^{-1}\left[\mathcal{F}[\delta\boldsymbol{M}] \cdot \tilde{\boldsymbol{k}}\right] \qquad \text{[S11]}$$

Note that the cross correlation in [11] is not the vector cross correlation. The function which this term is the autocorrelation of can then be rewritten:

$$\mathcal{F}^{-1}\left[\mathcal{F}[\delta\boldsymbol{M}] \cdot \tilde{\boldsymbol{k}}\right] = \delta\boldsymbol{M} \,\overline{*}\, \mathcal{F}^{-1}[\tilde{\boldsymbol{k}}] \qquad \text{[S12]}$$

To understand this term, we need the inverse Fourier transform of the unit vector $\tilde{\boldsymbol{k}}$. Let us denote this function $\mathcal{F}_{\tilde{\boldsymbol{k}}}^{-1}(\boldsymbol{r})$

$$\mathcal{F}_{\tilde{\boldsymbol{k}}}^{-1}(\boldsymbol{r}) = \mathcal{F}^{-1}\left[\tilde{\boldsymbol{k}}\right] = \frac{1}{(2\pi)^3} \int d\boldsymbol{k} \frac{\boldsymbol{k}}{|\boldsymbol{k}|} \exp(-i\boldsymbol{k} \cdot \boldsymbol{r}) \qquad \text{[S13]}$$

To solve this integral, we first look for symmetry in the integral. If we rotate the argument with operator R and use $\cdot R\boldsymbol{r} = R^{-1}\boldsymbol{k} \cdot \boldsymbol{r}$, $R^{-1}R = 1$, $|R^{-1}\boldsymbol{k}| = |\boldsymbol{k}|$ and $d\boldsymbol{k} = d(R^{-1}\boldsymbol{k})$ since the Jacobian of a rotation is 1, then we get:

$$\mathcal{F}_{\tilde{\boldsymbol{k}}}^{-1}(R\boldsymbol{r}) = \frac{1}{(2\pi)^3} \int d\boldsymbol{k} \frac{\boldsymbol{k}}{|\boldsymbol{k}|} \exp(-i\boldsymbol{k} \cdot R\boldsymbol{r}) = \frac{1}{(2\pi)^3} R \int d(R^{-1}\boldsymbol{k}) \frac{R^{-1}\boldsymbol{k}}{|R^{-1}\boldsymbol{k}|} \exp(-iR^{-1}\boldsymbol{k} \cdot \boldsymbol{r}) = R\mathcal{F}_{\tilde{\boldsymbol{k}}}^{-1}(\boldsymbol{r})$$

[S14]

The value of the integral thus rotates in the same way as the position.

Next we look at the integrals behavior when scaling $\boldsymbol{r}$ with $t > 0$. Here we use that $\frac{\boldsymbol{k}}{|\boldsymbol{k}|} = \frac{t\boldsymbol{k}}{|t\boldsymbol{k}|}$ and $d(t\boldsymbol{k}) = t^3 d\boldsymbol{k}$ to get:

$$\mathcal{F}_{\tilde{k}}^{-1}(t\boldsymbol{r}) = \frac{1}{(2\pi)^3}\int d\boldsymbol{k}\frac{\boldsymbol{k}}{|\boldsymbol{k}|}\exp(-i\boldsymbol{k}\cdot t\boldsymbol{r}) = \frac{1}{(2\pi)^3}t^{-3}\int d(t\boldsymbol{k})\frac{t\boldsymbol{k}}{|t\boldsymbol{k}|}\exp(-it\boldsymbol{k}\cdot\boldsymbol{r}) = t^{-3}\mathcal{F}_{\tilde{k}}^{-1}(\boldsymbol{r})$$

[S15]

The length of the value of the integral thus scales as $|\boldsymbol{r}|^{-3}$.

As we now know how the integral behaves under rotation and scaling of $\boldsymbol{r}$, we now only need to solve the integral at two points, one being zero, and then use the found symmetry to get the full $\boldsymbol{r}$-dependence. We choose $\boldsymbol{r}_0 = (0,0,1)$:

$$\mathcal{F}_{\tilde{k}}^{-1}(\boldsymbol{r_0}) = \frac{1}{(2\pi)^3}\int d\boldsymbol{k}\frac{\boldsymbol{k}}{|\boldsymbol{k}|}\exp(-i\boldsymbol{k_3}) \quad \text{[S16]}$$

Changing to polar coordinates:

$$\mathcal{F}_{\tilde{k}}^{-1}(\boldsymbol{r_0}) = \frac{1}{(2\pi)^3}\int_0^\infty k^2 dk \int_0^\pi \sin\theta\, d\theta \int_0^{2\pi} d\phi \begin{pmatrix}\sin\theta\cos\phi\\ \sin\theta\sin\phi\\ \cos\theta\end{pmatrix}\cdot\exp(-i\cos\theta\cdot k) \quad \text{[S17]}$$

The ϕ and θ parts are easily solved, leaving us with:

$$\mathcal{F}_{\tilde{k}}^{-1}(\boldsymbol{r_0}) = \frac{i}{2\pi^2}\int_0^\infty dk \begin{pmatrix}0\\0\\1\end{pmatrix}\cdot(k\cdot\cos k - \sin k) \quad \text{[S18]}$$

This is solved by taking the limit:

$$\mathcal{F}_{\tilde{k}}^{-1}(\boldsymbol{r_0}) = \frac{i}{2\pi^2}\lim_{a\to 0}\int_0^\infty dk \begin{pmatrix}0\\0\\1\end{pmatrix}\cdot\exp(-ak)\,(k\cdot\cos k - \sin k) \quad \text{[S19]}$$

By rewriting cos and sin using exponentials and using $\int_0^\infty x^n \exp(-ax)\,dx = \frac{\Gamma(n+1)}{a^{n+1}}$, the integral is solved and the limit $a \to 0$ taken, giving:

$$\mathcal{F}_{\tilde{k}}^{-1}(\boldsymbol{r_0}) = \begin{pmatrix}0\\0\\-\frac{i}{\pi^2}\end{pmatrix} \quad \text{[S20]}$$

For $\boldsymbol{r} = (0,0,0)$ the θ part of the integral is easily seen to give the zero vector, $\boldsymbol{0}$.

Using the symmetry properties we can now write the general form of the integral as:

$$\mathcal{F}_{\widetilde{\boldsymbol{k}}}^{-1}(\boldsymbol{r}) = \mathcal{F}^{-1}[\widetilde{\boldsymbol{k}}] = \begin{cases} -\frac{i}{\pi^2}\frac{\boldsymbol{r}}{|r|^4}, & |r| \neq 0 \\ \boldsymbol{0}, & |r| = 0 \end{cases} \quad \text{[S21]}$$

It is more convenient to move all constant outside of the function and define

$$\boldsymbol{\Upsilon}(\boldsymbol{r}) = \begin{cases} \frac{\boldsymbol{r}}{|r|^4}, & |r| \neq 0 \\ \boldsymbol{0}, & |r| = 0 \end{cases} \quad \text{[S22]}$$

The expression for the 3D-mΔPDF can now be written, remembering that one term in the cross correlation is complex conjugate:

$$3D - m\Delta PDF = \frac{r_0^2}{4\mu_B^2}\langle \delta\boldsymbol{M} \overline{\otimes} \delta\boldsymbol{M} - \frac{1}{\pi^4}(\delta\boldsymbol{M} \mathbin{\overline{*}} \boldsymbol{\Upsilon}) \otimes (\delta\boldsymbol{M} \mathbin{\overline{*}} \boldsymbol{\Upsilon})\rangle \quad \text{[S23]}$$

S3.2. Elastic and energy-integrated scattering

The equations above are given in the static approximation where the scattered signal is integrated in energy over the magnetic excitations. In that case we get the equal-time correlation function. In the case where only elastic scattering is used, the equations have a small modification.

The cross section, before given by equation [S1] is for elastic only scattering instead given by (Lovesey, 1984)

$$\frac{d\sigma}{d\Omega} = r_0^2\langle \boldsymbol{Q}_\perp(-\boldsymbol{k})\rangle \cdot \langle \boldsymbol{Q}_\perp(\boldsymbol{k})\rangle \quad \text{[S24]}$$

The result of this in that the equation for the 3D-mΔPDF instead becomes

$$3D - m\Delta PDF = \frac{r_0^2}{4\mu_B^2}\langle \delta\boldsymbol{M}\rangle \overline{\otimes} \langle \delta\boldsymbol{M}\rangle - \frac{1}{\pi^4}(\langle \delta\boldsymbol{M}\rangle \mathbin{\overline{*}} \boldsymbol{\Upsilon}) \otimes (\langle \delta\boldsymbol{M}\rangle \mathbin{\overline{*}} \boldsymbol{\Upsilon}) \quad \text{[S25]}$$

Which is the same as before, but for the time averaged magnetization density.

In cases where the system does not have significant magnetic excitations such as magnons, the two equations are the same, as the time-average makes no difference.

This is the case for bixbyite, where the magnetic diffuse scattering is elastic. This can be seen in Figure S4 where the total energy integrated scattering for bixbyite is shown for the data before the elastic discrimination is used for 300K and 7K. It is seen that the magnetic signal which appears at 7K is the same as was seen in figure 3, where the elastic-only contribution is shown.

The two cases, equation [S23] and [S25] are two extremes where full energy integration or purely elastic scattering is used. In practice all experiments have a finite width for the energy integration. This integration width determines which modes are time-averaged in the 3D-mΔPDF, as in equation [S25] and which give an equal-time correlation as in equation [S23]. All modes with higher energy than the integration width will be averaged in the resulting 3D-mΔPDF.

The CORELLI instruments elastic resolution changes with the scattering vector, and the resolution is better for short scattering vectors than long. The scattering vector dependency of the energy resolution is discussed by (Ye et al., 2018), where they find the energy resolution to range from 0.4 to 2.5 meV for a collected dataset.

Table S1 Structure refinement results from single-crystal neutron diffraction data for bixbyite.

Temperatures [K]	7	25	50	80	160	240	300
Unit cell length [Å]	9.409(1)	9.409(1)	9.399(1)	9.402(1)	9.401(1)	9.411(1)	9.401(1)
24*d* site Mn occupancy	0.508(3)	0.508(3)	0.506(4)	0.508(3)	0.507(3)	0.509(3)	0.510(4)
8*b* site Mn occupancy	0.243(6)	0.245(5)	0.247(6)	0.250(5)	0.247(5)	0.244(5)	0.245(6)
24*d* site z-coordinate	0.4585(1)	0.45831(1)	0.45833(2)	0.45831(1)	0.45822(1)	0.45808(1)	0.45829(2)
Oxygen x-coordinate	0.61514(7)	0.61512(7)	0.61517(8)	0.61517(7)	0.61519(8)	0.61519(7)	0.61531(8)
Oxygen y-coordinate	0.83768(6)	0.83771(7)	0.83771(7)	0.83771(7)	0.83769(7)	0.83767(7)	0.83767(7)
Oxygen z-coordinate	0.60997(7)	0.60998(7)	0.61001(8)	0.60996(7)	0.60994(7)	0.60990(7)	0.60983(7)
Component of first twin	0.522(4)	0.458(4)	0.473(4)	0.455(4)	0.459(4)	0.430(4)	0.466(4)
Goodness of Fit	1.250	1.195	1.210	1.183	1.219	1.220	1.251
R1	0.0405	0.0426	0.0454	0.0441	0.0429	0.0418	0.0428
wR	0.1097	0.1094	0.117	0.113	0.1127	0.1061	0.1161

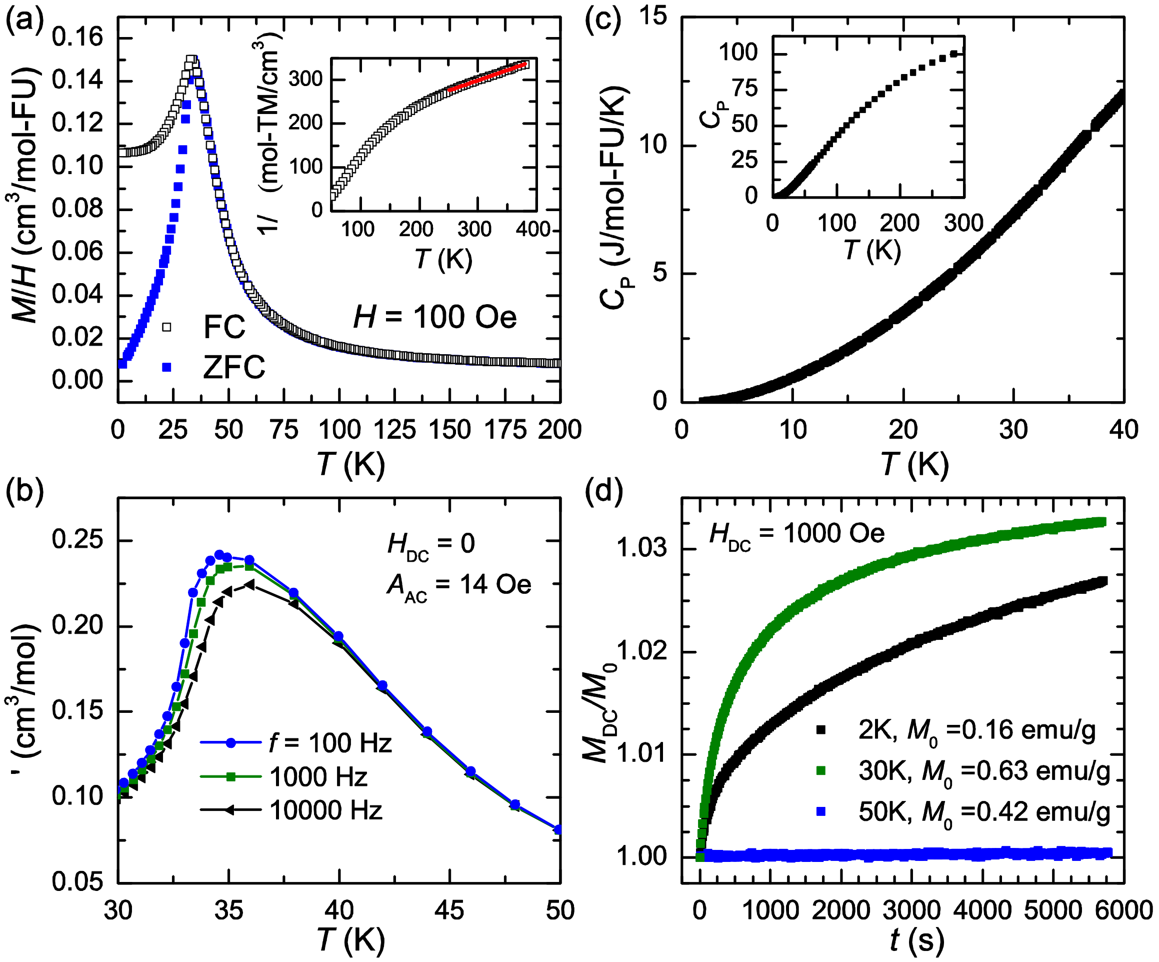

Figure S1 Physical property measurements on bixbyite. (a) Temperature-dependent magnetization data for field-cooled (FC) and zero field cooled (ZFC) measurements showing a divergence between the two datasets. (b) in-phase component of the ac susceptibility showing frequency dependence of the broad peak near 32.5K (c) Specific heat capacity data do not reveal any clear anomaly associated with the magnetic transition near 32.5K. (d) Time-dependence of the dc magnetization data at various temperatures demonstrating glassy dynamics below the freezing temperature.

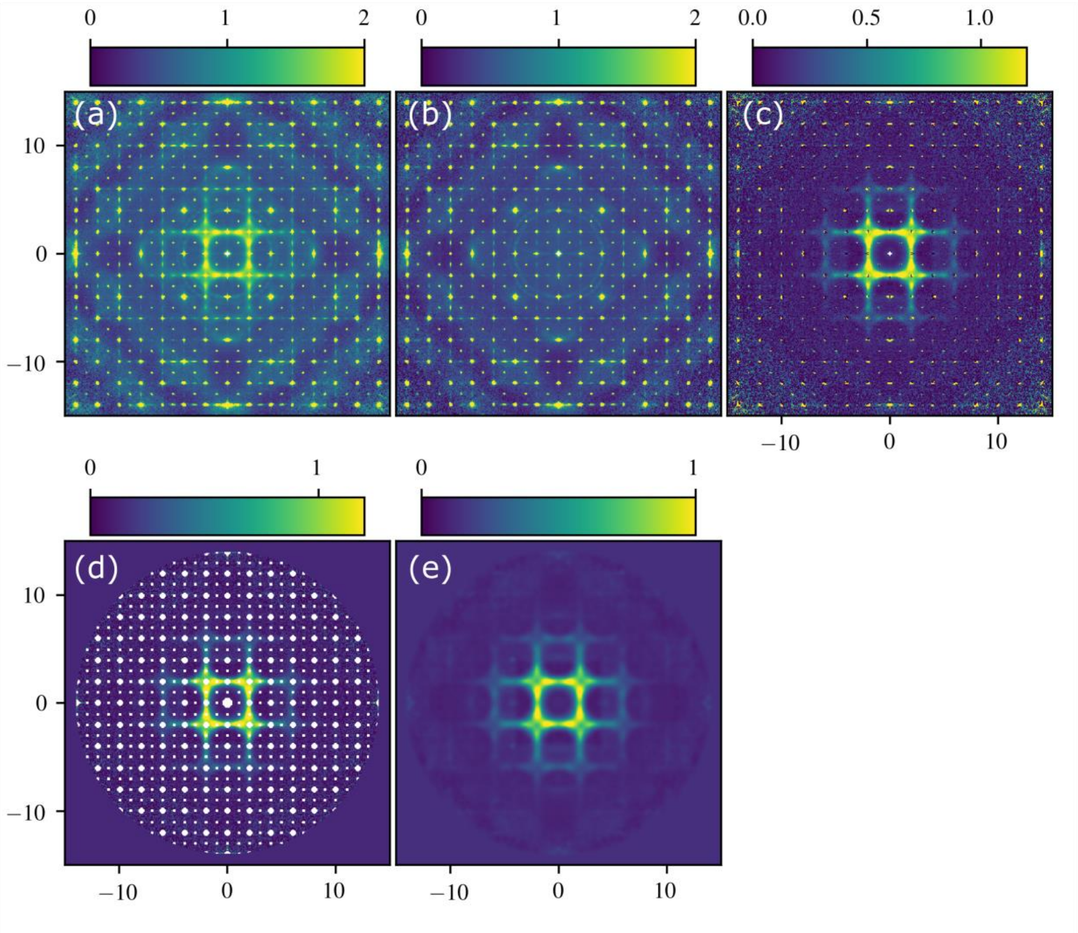

Figure S2 Process from scattering data to isolated magnetic diffuse scattering. (a) Total elastic neutron scattering at 7K. (b) Total elastic neutron scattering at 300K. (c) Difference scattering obtained by subtracting the 300K data from the 7K data. (d) Residual intensities at Bragg positions and high angle noise removed. (e) Isolated magnetic diffuse scattering after filling in the removed Bragg areas with a smooth function resembling the diffuse scattering.

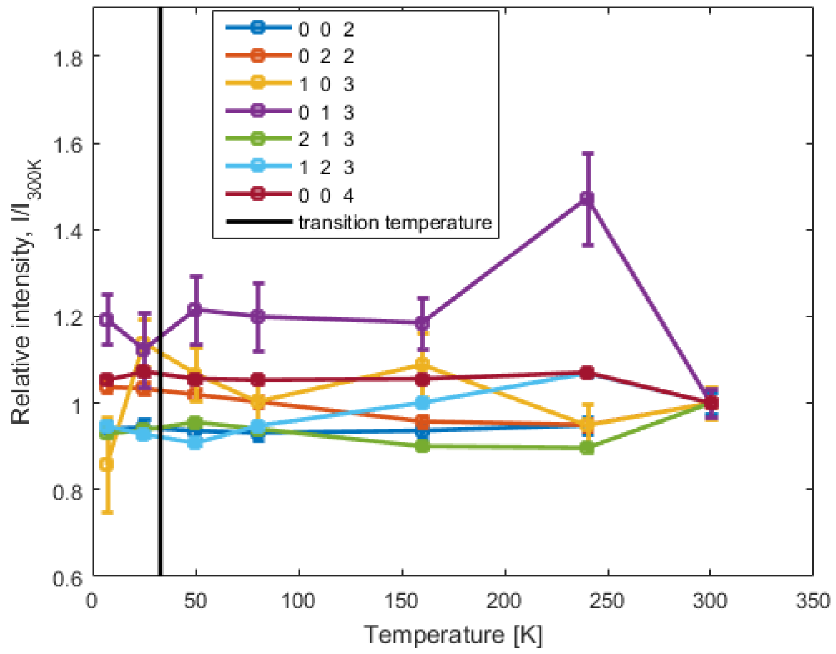

Figure S3 **Normalized low order reflection intensities as a function of temperature**. No systematic changes are seen at the transition temperature (Vertical black line.)

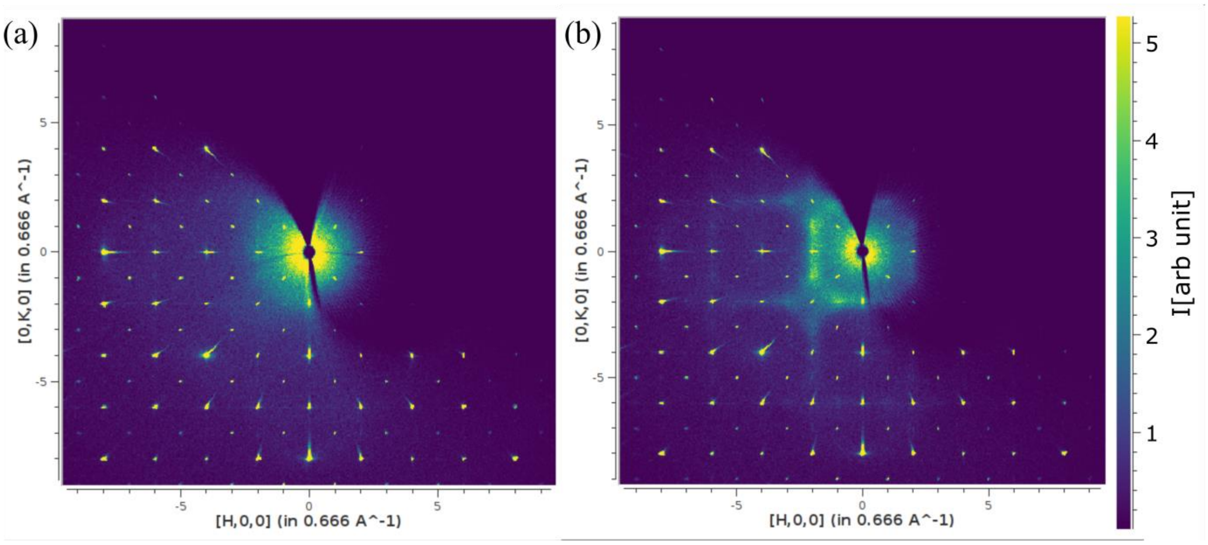

Figure S4 Energy integrated scattering from bixbyite before corrections and elastic-only discrimination for (a) 300K and (b) 7k. The magnetic diffuse scattering appearing at 7K is the same as was seen in figure 3 where the elastic component is shown.

S4. Additional References

Balanda, M. (2013). *Acta Phys Pol A* **124**, 964-976.

Binder, K. & Young, A. P. (1986). *Rev Mod Phys* **58**, 801-976.

Ye, F., Liu, Y., Whitfield, R., Rozenkranz, S., Osborn, R. (2018) submitted to J. Appl. Cryst.

Kobas, M., Weber, T. & Steurer, W. (2005). *Phys Rev B* **71**,

Mydosh, J. A. (1993). *Spin glasses : An experimental introduction*. London ; Washington, DC: Taylor & Francis.

Robitaille, T. P., Tollerud, E. J., Greenfield, P., Droettboom, M., Bray, E., Aldcroft, T., Davis, M., Ginsburg, A., Price-Whelan, A. M., Kerzendorf, W. E., Conley, A., Crighton, N., Barbary, K., Muna, D., Ferguson, H., Grollier, F., Parikh, M. M., Nair, P. H., Guenther, H. M., Deil, C., Woillez, J., Conseil, S., Kramer, R., Turner, J. E. H., Singer, L., Fox, R., Weaver, B. A., Zabalza, V., Edwards, Z. I., Bostroem, K. A., Burke, D. J., Casey, A. R., Crawford, S. M., Dencheva, N., Ely, J., Jenness, T.,

Labrie, K., Lim, P. L., Pierfederici, F., Pontzen, A., Ptak, A., Refsdal, B., Servillat, M., Streicher, O. & Collaboration, A. (2013). *Astron Astrophys* **558**,

Sheldrick, G. M. (2001). Bruker-AXS, Madison, Wisconsin, USA.

Sheldrick, G. M. (2008). *Acta Crystallographica Section A* **64**, 112-122.
